# Evaluation of Major Minerals and Trace Elements in Wild and Domesticated Edible Herbs Traditionally Used in the Mediterranean Area

**DOI:** 10.1007/s12011-020-02467-3

**Published:** 2020-11-05

**Authors:** Costanza Ceccanti, Andrea Brizzi, Marco Landi, Luca Incrocci, Alberto Pardossi, Lucia Guidi

**Affiliations:** 1 Department of Agriculture, Food and Environment, University of Pisa, Pisa, Italy; 2 Interdepartmental Research Center Nutrafood “Nutraceuticals and Food for Health”, University of Pisa, Pisa, Italy

**Keywords:** Human diet, Major minerals, Trace elements, Wild edible herbs, Domestication

## Abstract

The human diet is characterized by the intake of major minerals (Na, K, Ca, Mg, P, N) and trace elements (Zn, Mn, Se, Cu, Fe, Co, I, Cr, F, Pb, Cd) for their key role in many metabolic functions. Nowadays, the research of sources able to improve their intake is in continuous evolution, especially in the undeveloped countries. In this sense, wild edible herbs, commonly used since ancient times, can represent a good alternative to improve the daily human intake of minerals. In this study, four wild edible species, *Rumex acetosa*, *Picris hieracioides*, *Cichorium intybus*, and *Plantago coronopus*, were analyzed for their content in Na, K, Ca, Mg, Cu, Mn, Fe, and Zn and, besides, three domestications (named “soilless,” pot, and open field) were evaluated in the analyzed species in the prospective of their commercialization as valuable sources of minerals in the human diet. Nitrate and oxalate contents were also evaluated, given their negative impact on human health. Results unveil that open field domestication allowed the plants to maintain the content of major minerals similar to those measured in wild plants, especially in *C. intybus* and *P. hieracioides*. The trace elements Cu, Mn, Fe, and Zn were not recorded at high content irrespectively to the wild collection or domestications. Finally, plants grown in the open field also accounted for a high oxalate and nitrate content, especially in *R. acetosa.* Further researches should be aimed at decreasing the oxalate and nitrate content in the domesticated species and to promote the commercialization of the domesticated species.

## Introduction

The quantification of major minerals for human diet (sodium, potassium, calcium, magnesium, phosphorus) and trace elements (zinc, manganese, selenium, copper, iron, cobalt, iodine, chromium, fluorine, lead, cadmium) in leaves from different wild edible herbs, the research about their roles in various human metabolic processes, and, therefore, their impact on human health have an increased interest due to mineral and trace element deficiencies in the human diet [1]. In fact, these micronutrients are largely present in wild species used as dietary item as well as ingredient in traditional recipes of the Mediterranean area [2].

Nowadays, a serious global problem in the human diet has become trace element deficiencies, especially in areas where the diet lacks variety [3]. For example, iron has been recognized as the most diffuse form of trace element affliction, while the prevalence of zinc deficiency is also thought to be relevant [1]. Iron deficiency and low hemoglobin can cause iron deficiency anemia, infectious disorders, and hemoglobinopathies. Dietary intake of iron is low in populations consuming monotonous plant-based diet [4]. The most important strategy to decrease trace element deficiencies has been applied to address dietary modifications and variations, focusing on increasing the amount of trace elements consumed in the diet and, simultaneously, maintaining a balanced intake of minerals [3].

Currently, the modernization and the globalization in agriculture have resulted in simplification of diets with the imposition of few staple crops [5]. For this reason, the integration of wild edible herbs to human diets has been promoted; an increasing consensus that wild species could significantly contribute in alleviating malnutrition and hunger and, consequently, neglected crops and wild edible herbs are receiving renewed attention, with the identification of them as convenient sources of income and vehicles for improved nutrition [1]. Little researches are already available about mineral and trace element composition in domesticated and wild edible herbs; this is probably because the uptake of these components strongly depends on botanical and genetic characteristics, besides different environmental and agronomic conditions [6–9]. For example, Disciglio et al. [6] showed that wild rocket (*Diplotaxis tenuifolia* (L.) DC.) contained higher value of sodium, potassium, and fluoride than cultivated rocket and Turan et al. [7] reported that 26 wild edible species contained higher value of N, K, Ca, and Mg than cultivated lettuce, spinach, or cabbage.

Besides their excellent major mineral and trace element amounts when compared to common leafy vegetables, wild edible herbs are often abundant in toxic compounds and they could not be used in the human diet or not eaten in large quantity. In fact, some studies reported a high content of oxalate and nitrate and other toxic compounds in *Rumex acetosa* L., *Sanguisorba minor* Scop., *Portulaca oleracea* L., and other wild edible species [10–12].

For all these reasons, a preliminary assessment of different domestications of the wild edible herbs should be investigated to promote the higher amount of major minerals and trace elements able to satisfy human diet requirement and to reduce possible high content of unhealthy elements such as nitrate or oxalate. The recognition of domestications able to obtain an efficient reproducibility of plants with a balanced micronutrient content represents a first step toward the large-scale commercialization of new edible species and, therefore, the variability of the human diet.

The aim of this study was to increase the knowledge about major minerals and trace elements in plants of four edible species, *Cichorium intybus*, *Rumex acetosa*, *Picris hieracioides* L., and *Plantago coronopus* L. (commonly used since ancient times during famine periods in mixtures, boiled or in soups in the Mediterranean area and already described in a previous study [2]) collected in the wild or subjected to three domestications (namely “soilless” (SS), pot (P), and open field (OF)). In particular, this preliminary investigation aimed at evaluating the domestications able to preserve micronutrient content, this in order to compensate for lack of major minerals and trace elements in the human diet and favoring the marketability of these “rediscovered” species.

## Materials and Methods

### Plant Material, Growing Conditions, and Experimental Design

Ten seedlings of *C. intybus*, *R. acetosa*, *P. hieracioides*, and *P. coronopus* were gathered in the wild (W) in the Pisa area as reported by Ceccanti et al. [13]. Furthermore, seedlings of the same species were cultivated using three different kinds of domestication: “soilless” (SS), pot (P), and open field (OF) systems. Details of different domestications were reported in a previous study [13].

Before the flowering, leaves of the domesticated and wild species were collected with a cut up above the substrate level, taking care to collect the youngest leaves of the basal rosette, botanical characteristic of all the examined species. Leaves of each species from wild collection or each different domestication were randomly selected, pooled, weighed, and oven-dried at 65 °C to constant weight and the dry weights were then noted, and percentage of dry matter (DM) was calculated. Dry samples were used for the chemical analyses that were carried out in triplicate.

### Mineral and Trace Element Analysis

A total of 200 mg dried samples were powdered and mineralized (90 min at 220 °C) using a solution of HNO_3_:HClO_4_ (2.5:1; v:v). Minerals (sodium, potassium, calcium, and magnesium) and some trace elements (copper, iron, manganese, and zinc) were determined using an atomic absorption spectrometer (Varian 240FS AA, Sidney, Australia). Data are expressed as mg mineral per g fresh weight (FW) or μg trace element per g FW.

### Nitrate and Oxalate Analysis

Nitrate (NO_3_^−1^) and oxalate (C_2_O_4_^−2^) concentrations were determined using an IonPac AG4A guard column and an IonPac AS4A analytical column of an ion exchange chromatograph (Dionex DX120, Dionex Corporation, Sunnyvale, CA, USA) with a conductivity detector as reported by D’Imperio et al. [14], with minor modifications. The eluent was constituted by 1.8 mM Na_2_CO_3_/1.7 mM NaHCO_3_ with a flow rate of 2 mL/min. The suppressor was an anion micromembrane suppressor (the AMMS-II) and the regenerant was constituted by 50 mN H_2_SO_4_ with a flow rate of 3–5 mL/min. The column storage solution was constituted by 100 mM NaOH and the background conductivity was 15–20 μS. Standard solutions of 5 ppm NaNO_3_ (Sigma Aldrich, Saint Louis, USA) and of 10 ppm CaC_2_O_4_ (Sigma Aldrich, Saint Louis, USA) were injected as reference solutions. The extraction was obtained by adding dried and powdered material (0.2 g) to 25 mL of Milli-Q water and incubating samples in a water bath, kept in agitation for 1 h at 60 °C. Data are expressed as mg nitrate or oxalate per g FW.

### Statistical Analysis

Data were subjected to a one-way ANOVA and statistical differences between wild and domesticated species under investigation were calculated by the least significant difference (LSD; *P* = 0.05) with GraphPad Software (GraphPad, La Jolla, USA). Data are expressed as mean ± standard deviation (± SD) of three replicates.

## Results and Discussion

### Major Minerals and Trace Elements

Table 1 reports the results of dry matter of the species under investigation harvested in wild conditions or after domestication with three agronomical practices.

**Table 1:** Influence of wild collection (W) or domestication (SS: “soilless”; P: pot; and OF: open field) on leaf dry matter (DM) of *Rumex acetosa*, *Picris hieracioides*, *Cichorium intybus*, and *Plantago coronopus*

Leaf DM (%)					
Species	Wild collection or cultivation technique				
	SS	P	OF	W	Significance
*Rumex acetosa*	7.0 ± 0.6b	5.5 ± 0.7c	10.9 ± 0.3a	10.3 ± 0.4a	***
*Picris hieracioides*	9.5 ± 1.3c	7.8 ± 0.5d	12.5 ± 0.2b	14.4 ± 0.5a	***
*Cichorium intybus*	8.8 ± 0.6c	9.3 ± 0.3bc	14.1 ± 0.8a	10.3 ± 1.0b	***
*Plantago coronopus*	7.8 ± 1.0c	5.8 ± 0.1d	10.9 ± 0.4a	9.5 ± 0.4b	***

Each value is the mean (± SD) of three replicates; values followed by the same letter within a row are not significantly different (*P* < 0.05). In the last column, the significance of the *F* ratio following one-way ANOVA test is reported. ***P < 0.001

DM accumulation was significantly higher in all the species collected as wild, even though *P. coronopus* and *C. intybus* DM was similar to plants gathered in the wild once domesticated in OF. The higher amount of DM measured in wild plants may be due to the advantage of nutrient solution supply in both SS and P domestications, which led plants to have a higher moisture content [15]. Our findings agree with results of Nicola et al. [15] who reported that rocket grown in a soil system had 41.5% higher DM content than plants grown in a soilless system. Moreover, our data are close to dry matter data reported by other researches (in a range of 7–29%) for different species of wild leafy vegetables [16, 17].

The concentrations of main major minerals Na, K, Ca, and Mg useful for human diet were analyzed in the four examined wild and domesticated edible species (Table 2). Significative differences among wild and domesticated plants were observed in all the species, except for the Na amount which was similar in both wild and domesticated plants in *P. coronopus*. In most of the cases, the OF domestication produced plants with foliar mineral contents similar to those measured in leaves of wild species. Some species maintained the mineral content of the major minerals during the SS domestication, e.g., the Na amount in *P. hieracioides*, or during the P domestication, such as the Na amount in *C. intybus*.

**Table 2:** Major mineral composition of *Rumex acetosa*, *Picris hieracioides*, *Cichorium intybus*, and *Plantago coronopus* leaves wild collected (W) and domesticated according to three different domestications: “soilless” (SS), pot (P), and organic field (OF)

Species	Wild collection or cultivation technique	Minerals (mg g^−1^ FW)			
		Na	K	Ca	Mg
*Rumex acetosa*	SS	0.22 ± 0.03b	3.03 ± 0.22bc	0.47 ± 0.06c	0.45 ± 0.03b
	P	0.11 ± 0.08c	2.82 ± 0.39c	0.72 ± 0.01b	0.51 ± 0.04b
	OF	0.13 ± 0.02c	5.76 ± 0.43a	1.72 ± 0.12a	1.20 ± 0.06a
	W	0.60 ± 0.04a	3.35 ± 0.10b	0.64 ± 0.02b	0.21 ± 0.01c
Significance		***	***	***	***
*Picris hieracioides*	SS	1.36 ± 0.12a	2.90 ± 0.30d	0.98 ± 0.07d	0.14 ± 0.03b
	P	1.30 ± 0.04a	3.62 ± 0.07c	1.50 ± 0.07c	0.06 ± 0.03c
	OF	0.74 ± 0.11c	7.32 ± 0.65a	3.57 ± 0.08b	0.54 ± 0.01a
	W	0.95 ± 0.04b	6.02 ± 0.06b	4.73 ± 0.65a	0.08 ± 0.05c
Significance		***	***	***	***
*Cichorium intybus*	SS	0.94 ± 0.07b	4.62 ± 0.36b	1.16 ± 0.07d	0.18 ± 0.02c
	P	1.58 ± 0.10a	3.70 ± 0.43c	1.52 ± 0.12b	0.33 ± 0.02b
	OF	0.41 ± 0.05c	10.95 ± 0.65a	2.53 ± 0.22a	0.51 ± < 0.01a
	W	1.59 ± 0.25a	4.05 ± 0.24bc	1.32 ± 0.10c	0.10 ± 0.01d
Significance		***	***	***	***
*Plantago coronopus*	SS	1.67 ± 0.32	2.82 ± 0.54b	1.60 ± 0.26b	0.14 ± 0.07bc
	P	1.30 ± 0.02	1.87 ± 0.20c	1.40 ± 0.07bc	0.18 ± 0.01b
	OF	1.72 ± 0.01	3.61 ± 0.19a	3.71 ± 0.07a	0.30 ± 0.01a
	W	1.27 ± 0.26	2.46 ± 0.27b	1.23 ± 0.11c	0.08 ± 0.05c
Significance		*ns*	**	***	**

Each value is the mean (± SD) of three replicates. Means within each column, considering each species separately, keyed with a different letter are significantly different at *P* = 0.05 and following, for each major mineral, one-way ANOVA with the different kinds of collection (SS; P; OF; W) as variability factor. For each species in the last row, the significance level of the *F* ratio following one-way ANOVA test is reported. ***P < 0.001; **P < 0.01; *ns*:not significant (*P* > 0.05)

The presence of minerals in the human diet is essential for the metabolism and the human health. For instance, K is fundamental in the maintenance of total body fluid volume and in various metabolic functions such as the protection of muscles and healthy effects on nerve activity [18, 19]. Potassium deficiency has been associated with hypertension as well as cardiovascular disease and stroke and the wild and OF-domesticated edible species under investigation reported K content similar to spinach cabbage and parsley (approximately 550 mg 100 g^–1^ FW), already considered as sources of this macronutrient [19]. The comparison of the examined species with common vegetables such as spinach, cabbage and parsley, and lettuce is necessary because the purpose of the study is to propose new edible species to enhance the major mineral and trace element intake in the human diet nowadays provided by other widely cultivated leafy vegetables.

Na is linked with K because of the competition of these two ions in human diet; therefore, an increasing intake of potassium in hypertensive subjects is recommended [20]. In fact, a reduced intake of Na is suggested because of its role in the hypertension disease [21]. The analyzed species showed higher level of Na than that found in common lettuce [22], even though, in some cases, the domestication resulted in decreasing the leaf Na level such as all the three domestications in *R. acetosa*, the OF domestication in *P. hieracioides*, and the OF and SS domestications in *C. intybus*.

Calcium is essential for the skeletal system and Ca deficiency leads to osteoporosis, which is a public health problem, especially in undeveloped countries [23]. The diet of the developed countries is characterized by dairy products rich in calcium (e.g., cheese, butter, yoghurt), but the cost of these foods is not affordable for most people living in undeveloped countries who, as a consequence, suffer of skeletal system diseases [23]. Accordingly, the species under investigation, especially those collected in the wild, might represent a reasonable source of calcium in the human diet, even though they are not able to improve the dairy calcium dose when compared with common vegetables such as lettuce and rocket [24, 25].

Magnesium is a fundamental constituent of bones and teeth; it is a cofactor for many enzymes in the human body such as ATP-dependent kinase and it affects permeability of membranes and neuromuscular transmission [26]. This mineral is a Ca antagonist on vascular smooth muscle tone and on post-receptor insulin signaling [27]. Nevertheless, our findings resulted lower than those found in wild and domesticated plants by Disciglio et al. [6], but, notably, higher than those found in lettuce cultivated using a soilless system by Fallovo et al. [24].

Table 3 reports the level of some trace elements in the species under investigation and the differences among wild and domesticated species. Leaves of the wild species showed the significantly highest Cu content, except for *C. intybus* in which the highest amount was recorded in OF-domesticated plants. Mn content was significantly higher in leaves of OF-domesticated plants than in wild plants. The only exception was represented by *R. acetosa* leaves in which Mn was similar in both P-domesticated and wild plants. Leaves of *P. hieracioides* reported the significative highest Fe content in leaves collected as wild between all analyzed domesticated samples in all species and leaves from *C. intybus* did not report significative differences between wild and domesticated plants. Furthermore, *R. acetosa* and *C. intybus* accumulated a higher level of Zn in leaves of SS- and P-domesticated plants when compared to wild-harvested plants. *P. hieracioides* reported similar Zn amount in wild plants and P-domesticated plants, while *P. coronopus* plants accounted for higher amount of Zn in SS-domesticated plants than in leaves collected from wild plants.

**Table 3:** Trace element composition of *Rumex acetosa*, *Picris hieracioides*, *Cichorium intybus*, and *Plantago coronopus* leaves wild collected (W) and domesticated according to three different domestications: “soilless” (SS), pot (P), and open field (OF)

Species	Wild collection or cultivation technique	Trace elements (μg g^−1^ FW)			
		Cu	Mn	Fe	Zn
*Rumex acetosa*	SS	1.91 ± 0.08b	7.95 ± 0.52b	15.76 ± 2.30c	8.93 ± 0.56a
	P	1.55 ± 0.03c	16.74 ± 3.72a	30.22 ± 6.69a	9.64 ± 0.93a
	OF	1.96 ± 0.15b	10.28 ± 0.18b	22.17 ± 2.66b	8.11 ± 1.03b
	W	2.86 ± 0.07a	14.96 ± 0.92a	22.73 ± 1.46b	6.85 ± 0.19c
Significance		***	**	*	**
*Picris hieracioides*	SS	1.95 ± 0.11c	9.75 ± 0.72c	20.22 ± 0.71c	8.77 ± 0.60b
	P	1.38 ± 0.21d	8.20 ± 0.69d	15.35 ± 0.62c	9.90 ± 1.35ab
	OF	3.45 ± 0.19b	16.71 ± 1.05a	39.21 ± 5.85b	7.20 ± 0.02c
	W	4.68 ± 0.58a	11.61 ± 0.36b	124.86 ± 12.82a	10.30 ± 0.50a
Significance		***	***	***	**
*Cichorium intybus*	SS	2.52 ± 0.11c	10.21 ± 0.21c	26.05 ± 1.75	11.09 ± 0.35a
	P	0.88 ± 0.03d	12.51 ± 1.30b	27.95 ± 12.18	10.95 ± 0.96a
	OF	4.16 ± 0.28a	18.19 ± 0.28a	39.74 ± 1.89	9.72 ± 1.32b
	W	2.86 ± 0.23b	7.07 ± 0.46d	24.62 ± 1.74	6.39 ± 0.57c
Significance		***	***	*ns*	***
*Plantago coronopus*	SS	1.61 ± 0.07c	7.69 ± 0.84a	34.19 ± 13.34a	8.35 ± 0.96a
	P	0.66 ± 0.13d	7.87 ± 1.20a	10.03 ± 2.91b	4.19 ± 1.13bc
	OF	1.88 ± 0.02b	7.66 ± 0.17a	18.30 ± 0.56ab	3.11 ± 0.74c
	W	2.68 ± 0.12a	4.77 ± 0.31b	28.55 ± 7.74a	4.53 ± 0.38b
Significance		***	**	*	***

Each value is the mean (± SD) of three replicates. Means within each column, considering each species separately, keyed with a different letter are significantly different at *P* = 0.05 and following, for each trace element, one-way ANOVA with the different kinds of collection (SS; P; OF; W) as variability factor. For each species in the last row, the significance level of the *F* ratio following one-way ANOVA test is reported. ***P < 0.001; **P < 0.01; *P < 0.05; *ns*:not significant (*P* > 0.05)

Cu, Mn, Fe, and Zn are considered as among the most important bivalent ions involved in human health and development [5]. Mn is a pivotal cofactor for many enzymes including the antioxidant enzyme Mn-superoxidase dismutase and the pyruvate carboxylase, as well as hydrolases, transferases, and kinases; some of them are important in the metabolism of macronutrients, in the bone and cartilage development [28]. Dietary Mn intake is decreasing in the developed countries because of the shifting of the human dietary intake to processed, fat, and sugary foods [28] and, therefore, the examined species could be useful in the developed countries. However, common vegetables, such as legumes or spinach, as well as medicinal herbs showed a Mn content close to the species examined in this study, even though the main sources of Mn in the diet result commonly from the whole grains [29–31]. Iron is an essential component of some proteins as Fe-heme proteins (e.g., hemoglobin, myoglobin, catalase, and cytochromes), proteins for Fe storage and transport (i.e., transferrin, hemosiderin, lactoferrin, and ferritin), and enzymes (e.g., aconitase, fumarate, reductase, NADH dehydrogenase, succinate dehydrogenase, cyclooxygenases, and alcohol dehydrogenases) [28]. It is also required for energy production [27]. Our findings resulted similar to those reported by Turan et al. [7] in edible plants collected as wild in Eastern Anatolia. Conversely, our results resulted lower than those reported in lettuce cultivated in open field and in greenhouse by Woolley et al. [32], showing higher Fe uptake in open field growing plants and those reported by Polat et al. [33] in organically growing iceberg lettuce.

Copper is an essential element in mammalian nutrition as a component of metalloenzymes. [34]. Cu is also necessary for the development of connective tissue and nerve covering and participates in the Fe metabolism [27]. The examined species had higher Cu levels if compared with lettuce [32, 35] and lower Cu levels if compared with medicinal herbs such as *Echinacea purpurea* [36]. In our analyses, it is not possible to figure out changes in Fe content observed between the different cultivation techniques. In fact, according to most of our findings, Woolley et al. [32] observed the decrease of trace elements in plants domesticated in greenhouse and an increase in those domesticated in open field.

Zinc is one of the major inhibitors of copper absorption in human diet, by competing with Cu for transport and by increasing intestinal metallothioneins, useful proteins in detoxification and metal buffering [28]. Moreover, Zn in the human health is involved in the activity of numerous enzymes and deficiency of this element (recorded especially in undeveloped countries) may compromise the immune system, wound healing, senses of taste and smell, and DNA synthesis [28]. The European Food Safety Authority (EFSA) suggested that an adequate intake (AI) for Zn is about 10 mg per day for a male adult, while the AI for Cu is 1.6 mg per day [37]. The examined edible species reported a medium-low Cu content, ranging from 4.1 to 26% (per a leaf portion of 100 g) of the daily AI and they had a low Zn content: 3.1 to 10.9% (per 100 g) of the Zn AI recommended by the EFSA [37]. The lower Zn amount suggests that this element is not available in the competition with Cu and the consumption of the species under investigation should be accompanied with meat or dairy products which are rich in Zn.

As reported for copper and zinc, the comparison between the AI of major minerals and trace elements suggested by the EFSA [37] and the percentage of AI may be useful to show the help that these “rediscovered” species might offer to the human diet. The wild species cannot be considered in this comparison as their commercialization would be difficult because of the inconstant growth and the scarce availability of these species into the wild. As defined by the EFSA, the AI is “the average observed daily level of intake by a population group (or groups) of apparently healthy people that is assumed to be adequate” [38]. Mineral and trace element requirements differ with age, sex, and physiological condition, due to differences in the velocity of growth for the younger age groups, and age-related changes in nutrient absorption and body functions and/or functional capacity, such as renal function [39]. In this study, the reported AI is related to adults and values are divided in male and female requirements (Table 4). Therefore, Table 4 reports the AI of mineral and trace elements analyzed in this study and the percentage contribution of the intake of 100 g of domesticated leaves to the AI of each element.

**Table 4:** Adequate intake (AI) of each examined mineral and trace element for male and female adults and the daily percentage contribution (%) of 100 g of *Rumex acetosa*, *Picris hieracioides*, *Cichorium intybus*, and *Plantago coronopus* leaves domesticated according to three different domestications: “soilless” (SS), pot (P), and open field (OF). When the value of AI was different between males and females, the daily percentage contribution was calculated on the value of males

				Daily percentage contribution of 100 g of plant											
		AI (mg day^−1^)		*R. acetosa*			*P. hieracioides*			*C. intybus*			*P. coronopus*		
	Age (years)	Male	Female	SS	P	OF	SS	P	OF	SS	P	OF	SS	P	OF
Na	≥ 18	2000	2000	1.1	0.5	0.6	6.8	6.5	3.7	4.7	7.9	2.0	8.3	6.5	8.6
K	≥ 18	3500	3500	8.6	8.0	16.4	8.3	10.3	20.9	13.2	10.6	31.3	8.0	5.3	10.3
Ca	≥ 25	750	750	6.3	9.6	22.9	13.1	20.0	47.6	15.5	20.3	33.7	21.3	18.7	49.5
Mg	≥ 18	350	300	12.8	14.6	34.3	4.0	1.7	15.4	5.1	9.4	14.6	4.0	5.1	8.6
Cu	≥ 25	1.6	1.3	11.9	9.7	12.2	12.2	8.6	21.5	15.7	5.5	26.0	10.1	4.1	11.7
Mn	≥ 18	3	3	26.5	55.8	34.3	32.5	27.3	55.7	34.0	41.7	60.6	25.6	26.2	25.5
Fe	≥ 18	6	7	26.3	50.4	36.9	33.7	25.6	65.3	43.4	46.6	66.2	56.9	16.7	30.5
Zn	≥ 18	10.1	8.2	8.8	9.5	8.0	8.7	9.8	7.1	10.9	10.8	9.6	8.3	4.1	3.1

Table 4 shows that the species under investigation may contribute to the balance of major mineral and trace element intake in the human diet, overcoming micronutrient deficiencies. Among domestications, the OF itinerary appeared the most efficient domestication method for major mineral maintenance (such as K, Ca, Mg), whereas trace element levels were generally maintained higher in P or SS domestications.

Trace element deficiencies are recognized as a serious global problem and since soilless systems are developed in controlled environment and substrates, in this preliminary study, P or SS domestications seem to be more efficient to alleviate trace element deficiency problems. However, further researches are necessary to confirm this aspect.

### Nitrate and Oxalate Content

Nevertheless, plants may be sources of toxic compounds such as nitrate, explaining the toxic effect in nitrite, and oxalate. Table 5 reports the level of oxalate and nitrate in the analyzed species and the differences among wild and domesticated species. The highest nitrate content was found in leaves from OF domestication in all the species under investigation, while the highest oxalate level was found in leaves of the species collected as wild, except in *R. acetosa* where the significative highest oxalate content was reported again in leaves from OF domestication.

**Table 5:** Nitrate and oxalate content of *Rumex acetosa*, *Picris hieracioides*, *Cichorium intybus*, and *Plantago coronopus* leaves wild collected (W) and domesticated according to three different domestications: “soilless” (SS), pot (P), and open field (OF)

Species	Wild collection or cultivation technique	Nitrate and oxalate (mg g^−1^ FW)	
		NO_3_	C_2_O_4_
*Rumex acetosa*	SS	< 0.01 ± < 0.01b	0.27 ± 0.03c
	P	0.07 ± 0.03b	1.40 ± 0.32b
	OF	0.34 ± 0.24a	8.50 ± 0.21a
	W	0.02 ± 0.01b	0.38 ± 0.14c
Significance		*	***
*Picris hieracioides*	SS	< 0.01 ± < 0.01c	< 0.01 ± < 0.01c
	P	1.18 ± 0.32b	0.05 ± 0.04b
	OF	2.58 ± 0.24a	< 0.01 ± < 0.01c
	W	0.13 ± 0.12c	0.18 ± 0.03a
Significance		***	***
*Cichorium intybus*	SS	< 0.01 ± < 0.01c	0.01 ± < 0.01c
	P	0.56 ± 0.56b	0.14 ± 0.02b
	OF	3.23 ± 0.30a	< 0.01 ± < 0.01c
	W	0.25 ± 0.10bc	0.18 ± 0.02a
Significance		***	***
*Plantago coronopus*	SS	< 0.01 ± < 0.01b	< 0.01 ± < 0.01b
	P	0.04 ± < 0.01b	< 0.01 ± < 0.01b
	OF	3.43 ± 0.32a	< 0.01 ± < 0.01b
	W	0.11 ± 0.05b	0.11 ± 0.01a
Significance		***	***

Each value is the mean (± SD) of three replicates. Means within each column, considering each species separately, keyed with a different letter are significantly different at *P* = 0.05 and following, for each anion, one-way ANOVA with the different kind of collection (S; P; O; W) as variability factor. For each species in the last row, the significance level of the *F* ratio following one-way ANOVA test is reported. ***P < 0.001; *P < 0.05

Nitrate content in vegetables is correlated to the risk factor for stomach cancer because of the nitrate ability to become nitrite in some specific conditions which, in turn, may link to hemoglobin, reducing the oxygen in human tissues, or to amines, forming cancerogenic amines named nitrosamines [40]. For these reasons, the EU Commission established maximum nitrate levels for the marketability of some vegetables such as spinach, lettuce, and rocket in the range between 2000 and 7000 mg kg^–1^ FW (Regulation No 1258/2011; https://eur-lex.europa.eu/legal-content/EN/TXT/PDF/?uri=CELEX:32011R1258&from=GA). Leaves of the species under investigation contained lower amount of nitrate than the established range and therefore are suitable for the commercialization, even though the OF domestication reported a higher nitrate content than 2000 mg kg^–1^ FW, but not exceeding 7000 mg kg^–1^ FW.

Besides, values of oxalate found in the leaves of *R. acetosa* were high, especially in OF domestication. Supportively, Yang and Loewus [41] reported values between 1.3 and 1.8 mg oxalate g^–1^ FW expressed as crude calcium oxalate in spinach, while the leaves of *R. acetosa* reported 8.5 mg oxalate g^–1^ FW in OF-grown plants. A high dietary oxalate intake stimulates secondary hyperoxaluria, a major risk factor for calcium oxalate stone formation [42]. An excessive oxalate intake also reduces the intestinal absorption of calcium and other trace elements, therefore impairing their bioavailability due to the formation of insoluble complexes [42].

## Conclusion

In this study, major minerals and trace elements useful for humans of four edible plants, *C. intybus*, *R. acetosa*, *P. hieracioides*, and *P. coronopus* (commonly used since ancient times in mixtures, boiled or in soups in the Mediterranean area), were determined in leaves once collected as wild, considering the nutritional and health roles of the analyzed minerals in the human diet. Afterwards, the preliminary investigation of an efficient domestication able to maintain mineral elements and trace elements was conducted. The main minerals K and Ca were at high level in leaves from plants domesticated in the OF, resulting sometimes higher accumulators than the wild species. Low content of trace elements was found in leaves of all species under investigation, as wild collected as well as domesticated, though *P. hieracioides* showed the highest Fe content when collected in the wild.

Finally, leaves of all the species from plants grown in the OF domestication reported the highest content of oxalate and nitrate, especially in those of *R. acetosa*. The presence of nitrate and oxalate is a negative factor in the human diet and their intake should be limited.

However, the OF domestication resulted the most promising method, maintaining the mineral content of the wild species, especially in *C. intybus* and *P. hieracioides*, resulting the most promising species for the intake of the main minerals in the human diet. Further researches are necessary to find suitable protocols of domestication aimed at decreasing the oxalate and nitrate content in the OF domestication and to make possible the commercialization of the analyzed species.
